# Quantifying *Wikipedia* Usage Patterns Before Stock Market Moves

**DOI:** 10.1038/srep01801

**Published:** 2013-05-08

**Authors:** Helen Susannah Moat, Chester Curme, Adam Avakian, Dror Y. Kenett, H. Eugene Stanley, Tobias Preis

**Affiliations:** 1 Department of Civil, Environmental and Geomatic Engineering, UCL, Gower Street, London, WC1E 6BT, UK; 2 Center for Polymer Studies, Department of Physics, Boston University, 590 Commonwealth Avenue, Boston, Massachusetts 02215, USA; 3 Warwick Business School, The University of Warwick, Scarman Road, Coventry, CV4 7AL, UK

## Abstract

Financial crises result from a catastrophic combination of actions. Vast stock market datasets offer us a window into some of the actions that have led to these crises. Here, we investigate whether data generated through Internet usage contain traces of attempts to gather information before trading decisions were taken. We present evidence in line with the intriguing suggestion that data on changes in how often financially related *Wikipedia* pages were viewed may have contained early signs of stock market moves. Our results suggest that online data may allow us to gain new insight into early information gathering stages of decision making.

The complex behaviour of our society emerges from decisions made by many individuals. In certain combinations, these numerous decisions can lead to sudden catastrophe, as demonstrated during crowd disasters and financial crises. Stock market data provide extremely detailed records of decisions that traders have made, in an area in which disasters have a widespread impact. As a result, these stock market records have generated considerable scientific attention^1^,^2^,^3^,^4^,^5^,^6^,^7^,^8^,^9^,^10^,^11^,^12^,^13^,^14^.

Human decision making does not, however, consist solely of the final execution of a chosen action, such as a trade recorded at a stock exchange. Instead, within the constraints of available resources, we often begin by gathering information to help us identify what the consequences of possible actions might be^15^.

With Internet provision becoming so widespread, online resources have become the first port of call in many quests for new information. As a rule, providers of such online resources collect extensive data on their usage, adding to a range of new large-scale measurements of collective human behaviour^16^,^17^,^18^,^19^,^20^,^21^. In this way, the ubiquity of the Internet in everyday life has not only changed the way in which people collect information to make decisions, but has opened up new avenues for scientists to investigate the early information gathering stages of decision making processes.

Previous studies have demonstrated that analysis of search data can provide insight into current or even subsequent behaviour in the real world. For example, changes in the frequency with which users look for certain terms on search engines such as *Google* and *Yahoo!* have been correlated with changes in the numbers of reports of flu infections across the USA^22^, the popularity of films, games and music on their release^23^, unemployment rates^24^,^25^, tourist numbers^25^, and trading volumes in the US stock markets^26^,^27^. A recent study showed that Internet users from countries with a higher per capita gross domestic product (GDP) search for proportionally more information about the future than information about the past, in comparison with Internet users from countries with a lower per capita GDP^28^.

In work most closely related to the study presented here, Preis, Moat and Stanley outline an analysis of historic data which suggests that changes in search volume for financially relevant search terms can be linked to stock market moves^29^. A further study analysed data from *Twitter* and considered the emotions of traders, rather than their information gathering processes, suggesting that changes in the calmness of *Twitter* messages could be linked to changes in stock market prices^30^.

In this study, we investigate whether data on the usage of the popular online encyclopaedia *Wikipedia*^31^,^32^,^33^,^34^ can be linked to subsequent decisions made in the stock markets. Specifically, can we find any evidence that changes in the numbers of views or edits to articles relating to companies and other financial topics on *Wikipedia* may provide insight into the information gathering process of investors?

## Results

To investigate the relationship between changes in large-scale information gathering behaviour on *Wikipedia* and market participants' trading decisions, we consider data on how often pages on the English language *Wikipedia* have been viewed, and how often pages on the English language *Wikipedia* have been edited. *Wikipedia* entries can be both viewed and edited by any Internet user. Data on *Wikipedia* page views were downloaded from the online service *stats.grok.se*, and data on *Wikipedia* page edits were obtained by parsing the *Wikipedia* “Revision history” page associated to the article. We analyse data generated between 10^th^ December 2007, the earliest date for which *Wikipedia* views data are available from *stats.grok.se*, and 30^th^ April 2012.

We calculate two measures of *Wikipedia* user activity: the average number of page views and the average number of page edits that have taken place for a given *Wikipedia* page in week *t*, where we define weeks as ending on a Sunday. All names of *Wikipedia* pages used and further details on data pre-processing are provided in the Supplementary Information. To quantify changes in information gathering behaviour, we choose one measure of *Wikipedia* user activity *n*(*t*), either page view or page edit volume, and calculate the difference between the page view or page edit volume for week *t*, to the average page view or page edit volume for the previous Δ*t* weeks: Δ*n*(*t*, Δ*t*) = *n*(*t*) − *N*(*t* − 1, Δ*t*) with *N(t −* 1, Δ*t*) = (*n*(*t* − 1) + *n*(*t* − 2) + *…* + *n*(*t* − Δ*t*))/Δ*t*, where *t* is measured in units of weeks.

We begin our comparison of changes in *Wikipedia* usage to subsequent stock market movements in this historic data by implementing a hypothetical investment strategy that uses data on either *Wikipedia* page views or *Wikipedia* page edits to trade on the Dow Jones Industrial Average (DJIA), following the approach introduced by Preis, Moat, and Stanley^29^. In this hypothetical strategy, we sell the DJIA at the closing price *p*(*t* + 1) on the first trading day of week *t* + 1 if the volume of views or edits has increased in week *t* such that Δ*n*(*t*, Δ*t*) > 0. We then close the position by buying the DJIA at price *p*(*t* + 2) at the end of the first trading day of the following week *t* + 2. Note that mechanisms exist which make it possible to sell stocks on a financial market without first owning them. If instead the volume of views or edits has decreased or remained the same in week *t* such that Δ*n*(*t*, Δ*t*) ≤ 0, then we buy the DJIA at the closing price *p*(*t* + 1*)* on the first trading day of week *t* + 1, and sell the DJIA at price *p*(*t* + 2) at the end of the first trading day of the coming week *t* + 2 to close the position.

We calculate the cumulative return *R* of a strategy by taking the natural log of the ratio of the final portfolio value to the initial portfolio value. If we take a short position—selling at the closing price *p*(*t* + 1) and buying back at price *p*(*t* + 2)—then the change in the cumulative return *R* for a strategy is *log*(*p*(*t* + 1)) − *log*(*p*(*t* + 2)). If we take a long position—buying at the closing price *p*(*t* + 1) and selling at price *p*(*t* + 2)—then the change in the cumulative return *R* is *log*(*p*(*t* + 2)) − *log*(*p*(*t* + 1)). In this way, buy and sell actions have symmetric impacts on the cumulative return *R* of a strategy. In addition, we neglect transaction fees, since the maximum number of transactions per year when using this strategy is only 104, allowing one closing and one opening transaction per week. We note that inclusion of transaction fees would of course diminish any profit if this hypothetical strategy were to be used in the real world. However, this assumption does not have consequences for conclusions about the relationship between user activity on *Wikipedia* and movements in the DJIA.

We compare the returns from the *Wikipedia* data based strategies to the returns from a random strategy. In the random strategy, a decision is made each week to buy or sell the DJIA. The probability that the DJIA will be bought rather than sold is always 50%, and the decision is unaffected by decisions in previous weeks. This random strategy leads to no significant profit or loss. For the statistical comparisons reported in the following sections, we use 10,000 independent realisations of this random strategy for the period between 10^th^ December 2007 and 30^th^ April 2012. We find no evidence that the overall return from these 10,000 realisations is significantly positive or significantly negative (mean return = 0.0002, *V* = 25012353, *p* = 0.97, *α* = 0.05, two-tailed one-sample Wilcoxon signed rank test of symmetry of distribution of returns around 0). We use a non-parametric test to check this point, as the distribution of returns deviates significantly from the normal distribution (*D* = 0.1716, *p* < 0.001, *α* = 0.05, Kolmogorov-Smirnov test). Similarly, the remainder of the analyses of return distributions reported here also use non-parametric tests. Throughout the rest of the results, the cumulative returns *R* of all non-random strategies are stated in terms of standard deviations above or below the mean cumulative return of the random strategy.

### Views and edits of *Wikipedia* articles about companies listed in the DJIA

**Figure 1:**
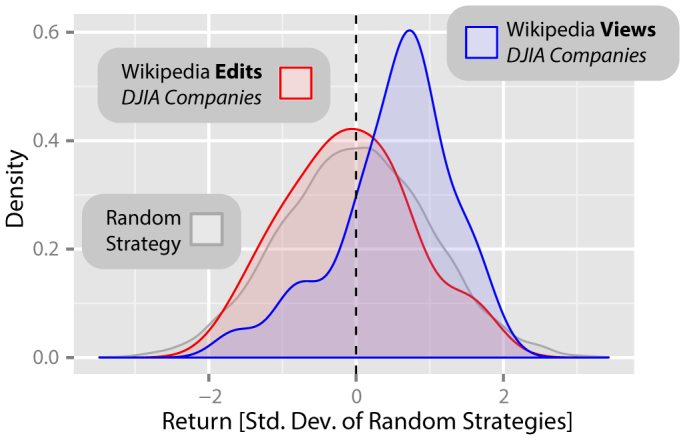
Returns from trading strategies based on *Wikipedia* view and edit logs for articles relating to the companies forming the Dow Jones Industrial Average (DJIA). The distributions of returns from two portfolios of 30 hypothetical strategies, trading weekly on the DJIA, based on changes in how often the 30 *Wikipedia* articles describing the companies listed in the DJIA were viewed (*blue*) and edited (*red*) during the period December 2007 – April 2012, with *Δt* = 3 weeks. The distribution of returns from 10,000 independent realizations of a random strategy is also shown (*gray*). Data is displayed using a kernel density estimate and the ggplot2 library^36^, with a Gaussian kernel and bandwidth calculated using Silverman's rule of thumb^37^. Whereas we show in the text that random strategies lead to no significant profit or loss, we find that the returns of *Wikipedia* article view based strategies for this period are significantly higher than the returns of the random strategies (mean *R* = 0.50; *W* = 199690, *p* = 0.005, *α* = 0.05, two-tailed two-sample Wilcoxon rank-sum test, Bonferroni correction applied). There is however no statistically significant difference between the returns from the *Wikipedia* edit based strategies and the random strategies (mean *R* = −0.09; *W* = 140781, *p* > 0.9, *α* = 0.05, two-tailed two-sample Wilcoxon rank-sum test, Bonferroni correction applied).

Figure 1 shows the distributions of returns from two portfolios of 30 hypothetical strategies, trading weekly on the DJIA. These trading strategies are based on changes in how often the 30 *Wikipedia* pages describing the companies in the DJIA were viewed (*blue*) and edited (*red*) during the period December 2007 – April 2012, with Δ*t* = 3 weeks. The distribution of returns from 10,000 independent realisations of a random strategy is also shown (*gray*).

We find that there are significant differences between these three distributions (*χ*^2^ = 10.21, *df* = 2, *p* = 0.006, *α* = 0.05; Kruskal-Wallis rank sum test). Our analysis shows that the returns of *Wikipedia* page view based strategies for this period are significantly higher than the returns of the random strategies (mean *R* = 0.50; *W* = 199690, *p* = 0.005, *α* = 0.05, two-tailed two-sample Wilcoxon rank-sum test, Bonferroni correction applied). There is however no statistically significant difference between the returns from the *Wikipedia* edit based strategies and the random strategies (mean *R* = −0.09; *W* = 140781, *p* > 0.9, *α* = 0.05, two-tailed two-sample Wilcoxon rank-sum test, Bonferroni correction applied).

### Views and edits of *Wikipedia* articles about financial topics

We investigate whether these results extend to *Wikipedia* articles on more general financial topics. To address this question, we make use of the fact that *Wikipedia* contains lists of pages relating to specific topics. Here, we examine view and edit data for 285 pages relating to general economic concepts, as listed in the subsection “*General economic concepts*” on the English language *Wikipedia* page “*Outline of economics*”.

**Figure 2:**
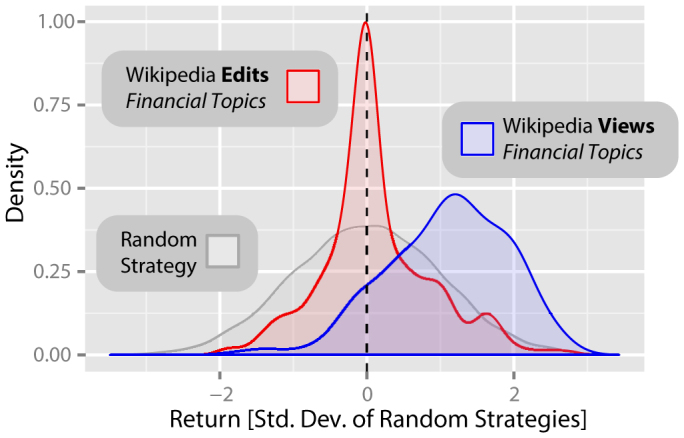
Returns from trading strategies based on *Wikipedia* access and edit logs for pages relating to finance. Parallel analysis of the distribution of returns from two much larger portfolios of 285 hypothetical strategies, based on changes in how often a set of 285 financially related *Wikipedia* pages were viewed (*blue*) and edited (*red*) during the same period as Figure 1, again with *Δt* = 3 weeks. Our analysis shows that the returns of *Wikipedia* page view based strategies are significantly higher than the returns of random strategies for this period (mean *R* = 1.10; *W* = 2286608, *p* < 0.001, *α* = 0.05, two-tailed two-sample Wilcoxon rank-sum test, Bonferroni correction applied). Once again however, we find no evidence of a statistically significant difference between the returns from the *Wikipedia* edit based strategies, and the random strategies (mean *R* = 0.12; *W* = 1516626, *p* = 0.19, *α* = 0.05, two-tailed two-sample Wilcoxon rank-sum test, Bonferroni correction applied).

Figure 2 shows the results of an analysis of the distribution of returns from two portfolios of 285 hypothetical strategies, trading weekly on the DJIA. These strategies are based on changes in how often these 285 financially related *Wikipedia* pages were viewed (*blue*) and edited (*red*) during the same period, again with Δ*t* = 3 weeks. As before, we find that there is a significant difference between the returns generated by the random strategies, the *Wikipedia* view based strategies and the *Wikipedia* edit based strategies (*χ*^2^ = 307.88, *df* = 2, *p* < 0.001, *α* = 0.05; Kruskal-Wallis rank sum test). As before, the returns of *Wikipedia* page view based strategies are significantly higher than the returns of random strategies for this period (mean *R* = 1.10; *W* = 2286608, *p* < 0.001, *α* = 0.05, two-tailed two-sample Wilcoxon rank-sum test, Bonferroni correction applied). Once again however, we find no evidence of a statistically significant difference between the returns from the *Wikipedia* edit based strategies, and the random strategies (mean *R = * 0.12; *W* = 1516626, *p* = 0.19, *α* = 0.05, two-tailed two-sample Wilcoxon rank-sum test, Bonferroni correction applied).

The lack of relationship found for the data on *Wikipedia* edits may simply reflect the substantial difference in the volume of data available for views and for edits, despite the much larger number of pages considered in this second analysis. For example, across the whole period, the *Wikipedia* articles on financial topics had an average of 1,351,796 views each, but only 431 edits. Of these pages, the most viewed page had 14,449,973 views, in comparison to 4832 edits. The least viewed page had 2,033 views, whereas 43 of the 285 pages in question had no edits at all. For the purposes of this study, we therefore do not consider edit data further.

### Strategy returns in different years

**Figure 3:**
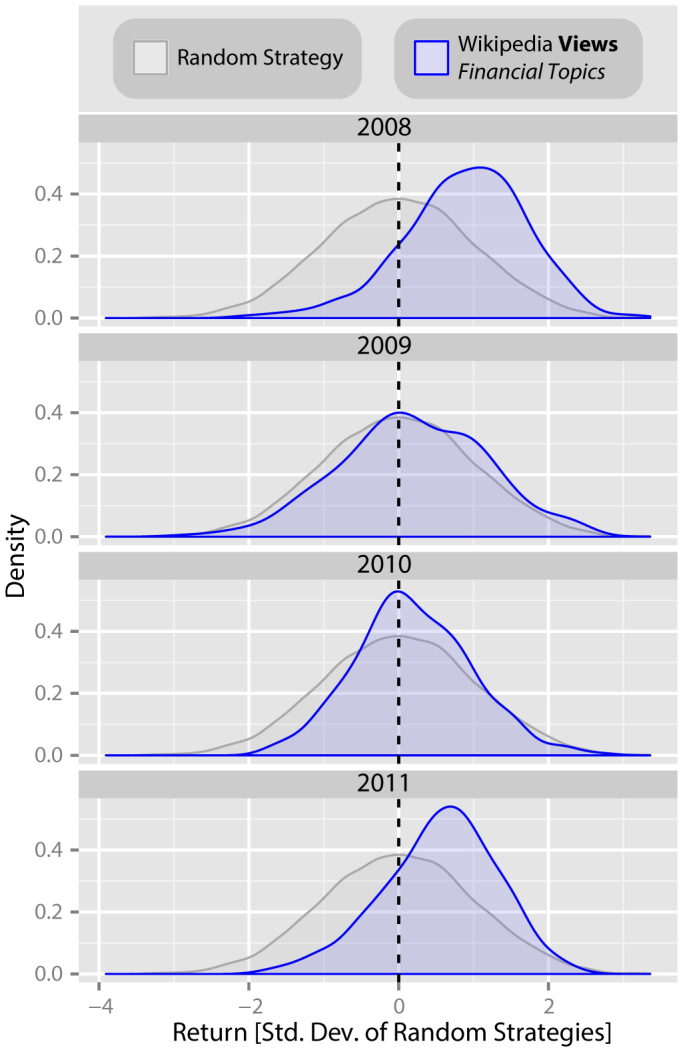
Yearly returns from trading strategies based on *Wikipedia* access logs for pages relating to finance. We investigate how returns from the trading strategies based on changes in views of the 285 financially related *Wikipedia* pages differ across time. The distribution of returns from the trading strategies, again with *Δt* = 3 weeks, are shown for each of the four years for which we have full *Wikipedia* page view data (*blue*) alongside returns from random strategies for that year (*grey*). Whilst returns differ from year to year (mean return for each year in standard deviations of random strategy returns for the given year: 2008, 0.89; 2009, 0.19; 2010, 0.19; 2011, 0.55; *χ*^2^ = 129.49, *df* = 3, *p* < 0.001; Kruskal-Wallis rank sum test), we find returns significantly greater than those of the random strategy for every 12 month period (2008: *W* = 2156094, *p* < 0.001; 2009: *W* = 1584915, *p* = 0.001; 2010: *W* = 1585336, *p* = 0.001; 2011: *W* = 1915511, *p* < 0.001; *α* = 0.05; all two-tailed two-sample Wilcoxon rank sum tests, using comparisons to the random strategy distributions for the given year).

The period of time we investigate here includes a particularly large drop in the DJIA in 2008. We therefore investigate what the returns from these trading strategies would have been for each individual year in our study period. Again, we consider the returns of strategies based on changes in views of the 285 financially related *Wikipedia* pages, again with Δ*t* = 3 weeks. In Figure 3, the distribution of returns from the trading strategies are shown for each of the four years for which we have full *Wikipedia* page view data (*blue*) alongside returns from random strategies for that year (*grey*).

We find that returns do differ from year to year (mean return for each year in standard deviations of random strategy returns for the given year: 2008, 0.89; 2009, 0.19; 2010, 0.19; 2011, 0.55; *χ*^2^ = 129.49, *df* = 3, *p* < 0.001; Kruskal-Wallis rank sum test). For every 12 month period however, we find returns significantly above those of the random strategy (2008: *W* = 2156094, *p* < 0.001; 2009: *W* = 1584915, *p* = 0.001; 2010: *W* = 1585336, *p* = 0.001; 2011: *W* = 1915511, *p* < 0.001; *α* = 0.05; all two-tailed two-sample Wilcoxon rank sum tests, using comparisons to the distribution of random strategy returns for the given year).

### The effect of Δ*t*

We investigate the effect of changes in Δ*t* on the returns from the trading strategies. Again, we consider portfolios of trading strategies based on changes in views of the 285 financially related *Wikipedia* pages*.* The mean return from trading strategies, expressed in standard deviations of random strategy returns, is shown in Figure S1 (see Supplementary Information) for Δ*t* = 1 to 10 weeks. We find that the mean return of the strategies does differ significantly for the different values of Δ*t* we tested (*χ*^2^ = 93.26, *df* = 9, *p* < 0.001; Kruskal-Wallis rank sum test). However, the mean return remains greater than 0 for all values of Δ*t* between 1 and 10 weeks (all *W*s > 1950000, all *p*s < 0.001; all two-tailed two-sample Wilcoxon rank sum tests, using comparisons to the random strategy distribution for the whole period).

### Mean return of the DJIA following increases and decreases in *Wikipedia* views

To complement the trading strategy analysis, we carry out a further analysis of weekly DJIA returns following increases and decreases in views of *Wikipedia* articles on financial topics.

For each of the 285 *Wikipedia* articles on financial topics, we identify all weeks *t* within our study period in which the volume of page views increased in week *t* such that Δ*n*(*t*, Δ*t*) > 0, using Δ*t* = 3. Across this set of weeks, we calculate the mean return of the DJIA during week *t* + 1, *log(p(t* + 2)) − *log(p(t* + 1)). Similarly, we calculate the mean return of the DJIA during week *t* + 1 for the set of weeks in which the volume of page views decreased in week *t* such that Δ*n*(*t*, Δ*t*) < 0.

Between these two sets of weeks, we find a significant difference in the mean return of the DJIA during week *t* + 1 (*W* = 78012, *p* < 0.001, *α* = 0.05, two-tailed two-sample Wilcoxon rank-sum test). Following a decrease in views of *Wikipedia* pages relating to financial topics, we find a mean DJIA weekly return of 0.0027 – a return significantly greater than 0 (*V* = 39592, *p* < 0.001, *α* = 0.05, two-tailed one-sample Wilcoxon signed rank test). In contrast, following an increase in views of *Wikipedia* pages relating to financial topics in week *t*, we find a mean DJIA weekly return of −0.0021, significantly less than 0 (*V* = 2222, *p* < 0.001, *α* = 0.05, two-tailed one-sample Wilcoxon signed rank test). The results of this analysis are therefore in line with the relationship between changes in views of *Wikipedia* articles on financial topics and subsequent movements in the DJIA suggested by the trading strategy analysis.

### Views and edits of *Wikipedia* articles about actors and filmmakers

Our assumption so far has been that only *Wikipedia* usage data relating to pages with financial connotations would provide any insight into information gathering processes before trading decisions, and therefore future changes in the DJIA. To verify this assumption, we carry out a further analysis of view data relating to 233 *Wikipedia* pages describing actors and filmmakers, as listed in the two subsections “*Featured articles*” and “*Good articles*” on the English language Wikipedia page “*Wikipedia:WikiProject Actors and Filmmakers*”. We suggest that such pages have less obvious financial connotations.

We analyse the distribution of returns for a portfolio of 233 hypothetical trading strategies based on changes in how often these pages were viewed, trading weekly on the DJIA with Δ*t* = 3 weeks for the same period as in previous analyses. We ensured that this set of pages, of similar size to the set of pages relating to financial topics, had at least equivalent traffic during the period of investigation, to ensure that any failure to find a relationship was not due to power issues caused through lack of data on *Wikipedia* views. Across the whole period, the actors and filmmakers pages had an average of 5,440,304 views each (in comparison to 1,351,796 for the financially related pages), where the least popular page had 2,261 views (in comparison to 2,033 views for the least popular financially related page) and the most popular page had 63,629,258 views (in comparison to 14,449,973 views for the most popular financially related page).

**Figure 4:**
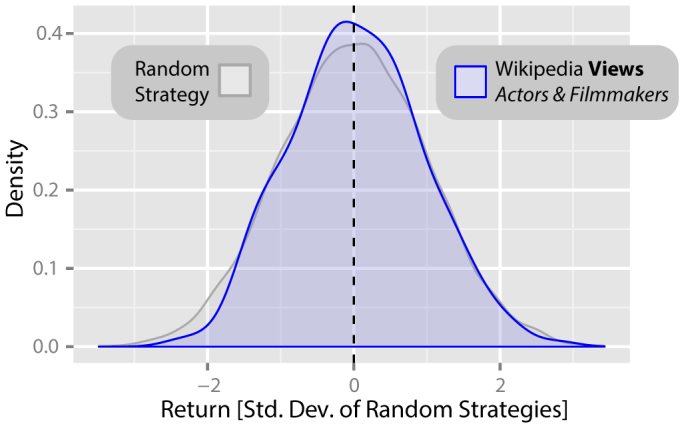
Returns from trading strategies based on *Wikipedia* access logs for pages relating to actors and filmmakers. Parallel analysis of the distribution of returns for another portfolio of 233 hypothetical strategies based on changes in how often a set of 233 *Wikipedia* pages relating to actors and filmmakers were viewed *(blue)*. Here, we find that there is no significant difference between the returns generated by the random strategies and the *Wikipedia* view based strategies (mean *R* = 0.04; *W* = 1189114, *p* = 0.59, *α* = 0.05, two-tailed two-sample Wilcoxon rank-sum test).

In Figure 4, we show the returns from these 233 strategies based on changes in the number of views of *Wikipedia* articles on actors and filmmakers (*blue*), alongside returns from the random strategies (*grey*). We find that there is no significant difference between the returns generated by the random strategies and the *Wikipedia* view based strategies (mean *R* = 0.04; *W* = 1189114, *p* = 0.59, *α* = 0.05, two-tailed two-sample Wilcoxon rank-sum test).

Similarly, for each of the 233 *Wikipedia* articles on actors and filmmakers, we calculate the return of the DJIA during week *t* + 1 for all weeks *t* where views of the article increased in comparison to views in the previous Δ*t* = 3 weeks such that Δ*n*(*t*, Δ*t*) > 0, and separately for all weeks *t* where views of the article decreased in comparison to views in the previous Δ*t* = 3 weeks such that Δ*n*(*t*, Δ*t*) < 0. We find no significant difference in the mean return of the DJIA during week *t* + 1 for these two sets of weeks (*W* = 28186, *p* = 0.47, *α* = 0.05, two-tailed two-sample Wilcoxon rank-sum test).

To summarise, neither an analysis based on the hypothetical trading strategy nor a complementary analysis of weekly DJIA returns find any evidence that changes in views of *Wikipedia* articles related to actors and filmmakers bear relation to future changes in the DJIA.

## Discussion

In summary, our results are consistent with the hypothesis that historic usage data from the online encyclopaedia *Wikipedia* between December 2007 and April 2012 may have provided some insight into future trends in the behaviour of financial market actors. In our analysis, we find evidence of increases in the number of page views of articles relating to companies or other financial topics before stock market falls. We do not, however, find any such relationship for changes in the weekly number of views of *Wikipedia* articles on the subject of actors and filmmakers, pages with less obvious financial connotations.

We propose one potential explanation in line with these results. We first suggest that *Wikipedia* records may provide a proxy measurement of the information gathering process of a subset of investors for the investigated period. We further note that previous studies in behavioural economics have demonstrated that humans are loss averse^35^: that is, they are more concerned about losing £5 than they are about missing an opportunity to gain £5. By this logic, it could be argued that the trading decision of greatest consequence for a trader would be to sell a stock at a lower price than they had previously believed it was worth. If we assume that investors may be willing to invest more efforts in information gathering before making a decision which they view to be of greater consequence, then it would follow that increases in information gathering would precede falls in stock market prices, in line with our results.

Our results suggest that Internet usage data may offer a window into the information gathering processes which precede actions captured in real world behaviour data sets. By combining these large data sets, we may be able to gain new insight into different stages of collective decision making.

## Author Contributions

H.S.M. and T.P. designed the study; H.S.M., C.C. and T.P. collected and analysed the data; and H.S.M., C.C., A.A., D.Y.K., H.E.S. and T.P. discussed the results and contributed to the text of the manuscript.

## Supplementary Material

Supplementary Information Supplementary Information: Quantifying Wikipedia Usage Patterns Before Stock Market Moves
