# Supplementary material for: Quantifying Wikipedia Usage Patterns Before Stock Market Moves
Source: Sci Rep. 2013 May 8;3:1801. doi: 10.1038/srep01801 (PMC3647164; doi:10.1038/srep01801)
Supplement: Supplementary Information — Quantifying Wikipedia Usage Patterns Before Stock Market Moves [file srep01801-s1.pdf]

TITLE:

Supplementary Information: Quantifying *Wikipedia* Usage Patterns Before Stock Market Moves

AUTHORS:

Helen Susannah Moat[1,2*], Chester Curme[2], Adam Avakian[2],
Dror Y. Kenett[2], H. Eugene Stanley[2], and Tobias Preis[3]

AFFILIATIONS:

[1] Department of Civil, Environmental and Geomatic Engineering, UCL,
Gower Street, London, WC1E 6BT, UK
[2] Department of Physics, Boston University,
590 Commonwealth Avenue, Boston, Massachusetts 02215, USA
[3] Warwick Business School, The University of Warwick,
Coventry, CV4 7AL, UK

* To whom correspondence should be addressed; E-mail: h.s.moat@ucl.ac.uk.

Data collected

Here we list the names of the *Wikipedia* pages for which we collected data on page views and edits in our study. All names relate to pages on the English *Wikipedia*, *http://en.wikipedia.org/*.

Pages relating to companies

This is a list of the *Wikipedia* pages relating to the 30 companies which formed the Dow Jones Industrial Average in April 2012.

View counts were downloaded from *http://stats.grok.se/* on 4 and 8 July 2012. Edit counts were retrieved directly from *Wikipedia* on 8 July.

1. *Alcoa*
2. *American Express*
3. *Boeing*
4. *Bank of America*
5. *Caterpillar Inc.*
6. *Cisco Systems*
7. *Chevron Corporation*
8. *DuPont*
9. *The Walt Disney Company*
10. *General Electric*
11. *The Home Depot*
12. *Hewlett-Packard*
13. *IBM*
14. *Intel*

15. *Johnson & Johnson*
16. *JPMorgan Chase*
17. *Kraft Foods*
18. *The Coca-Cola Company*
19. *McDonald's*
20. *3M*
21. *Merck & Co.*
22. *Microsoft*
23. *Pfizer*
24. *Procter & Gamble*
25. *AT&T*
26. *The Travelers Companies*
27. *United Technologies Corporation*
28. *Verizon Communications*
29. *Walmart*
30. *ExxonMobil*

Financially related pages

This is a list of the names of 285 pages relating to general economic concepts, as listed in the subsection "*General economic concepts*" on the English language *Wikipedia* page "*Outline of economics*". The list of terms was retrieved from the URL *http://en.wikipedia.org/wiki/Outline_of_economics* on 5 July 2012.

View counts were downloaded from *http://stats.grok.se/* on 6 July 2012. Edit counts were retrieved directly from *Wikipedia* on 8 July 2012.

1. *Agent (economics)*
2. *Aggregate demand*
3. *Aggregate supply*
4. *Agricultural policy*
5. *Antitrust*
6. *Arbitrage*
7. *Balance of trade*
8. *Big Mac Index*
9. *Big Push Model*
10. *Black market*
11. *Business cycle*
12. *Canadian and American economies compared*
13. *Capital (economics)*
14. *Capital asset*
15. *Capital intensity*
16. *Capitalism*
17. *Cartel*
18. *Cash crop*
19. *Catch-up effect*
20. *Central bank*
21. *Chicago school (economics)*
22. *Classical economics*
23. *Collective action*
24. *Collusion*
25. *Commodity*
26. *Commodity markets*
27. *Community currency*
28. *Comparative advantage*
29. *Competition*
30. *Complement good*

31. *Complementarity*
32. *Consumer*
33. *Consumer and producer surplus*
34. *Consumer price index*
35. *Consumer theory*
36. *Consumerism*
37. *Consumption (economics)*
38. *Cost*
39. *Cost-benefit analysis*
40. *Cost-of-living index*
41. *Cost-of-production theory of value*
42. *Currency*
43. *Debt*
44. *Decentralization*
45. *Deflation (economics)*
46. *Depression (economics)*
47. *Devaluation*
48. *Disinflation*
49. *Disposable income*
50. *Distribution (economics)*
51. *Economic*
52. *Economic data*
53. *Economic growth*
54. *Economic indicator*
55. *Economic profits*
56. *Economic reports*
57. *Economic subjectivism*
58. *Economic system*
59. *Economies of agglomeration*
60. *Economies of scale*

61. *Economies of scope*
62. *Economy*
63. *Ecosystem services*
64. *Efficiency wage hypothesis*
65. *Efficient market hypothesis*
66. *Elasticity (economics)*
67. *Employment*
68. *Entrepreneur*
69. *Entrepreneurship*
70. *Environmental finance*
71. *Euro*
72. *Event study*
73. *Experience economy*
74. *Export*
75. *Externality*
76. *Factor price equalization*
77. *Factors of production*
78. *Fair trade*
79. *Federal Reserve*
80. *Finance*
81. *Financial capital*
82. *Financial crisis*
83. *Financial instruments*
84. *Fiscal neutrality*
85. *Fiscal policy*
86. *Free goods*
87. *Free trade*
88. *Full-reserve banking*
89. *Game theory*
90. *General equilibrium*

91. *Globalization*
92. *Gold Standard*
93. *Good (economics)*
94. *Government-granted monopoly*
95. *Gross domestic product*
96. *Gross national product*
97. *History of economic thought*
98. *Home economics*
99. *Human Development Index*
100. *Human capital*
101. *Human development theory*
102. *Hyperinflation*
103. *Import*
104. *Import substitution*
105. *Incentive*
106. *Income*
107. *Income elasticity of demand*
108. *Income tax*
109. *Income velocity of money*
110. *Individual capital*
111. *Induced demand*
112. *Industrial Organization*
113. *Industrial Revolution*
114. *Industrial policy*
115. *Industrialisation*
116. *Inferior goods*
117. *Inflation (economics)*
118. *Input-output model*
119. *Interest*
120. *International trade*

121. *Investment*
122. *Investment policy*
123. *Invisible Hand*
124. *John Maynard Keynes*
125. *Keynesian economics*
126. *Knowledge-based economy*
127. *Labor market*
128. *Labor theory of value*
129. *Laissez-faire*
130. *Land (economics)*
131. *Land value tax*
132. *List of recessions*
133. *List of scholarly journals in economics*
134. *Living wage*
135. *Local currency*
136. *Local purchasing*
137. *Lorenz curve*
138. *Macroeconomics*
139. *Marginal Revolution*
140. *Marginalism*
141. *Market*
142. *Market (economics)*
143. *Market economy*
144. *Market failure*
145. *Market form*
146. *Market power*
147. *Market share*
148. *Market system*
149. *Market transparency*
150. *Means of production*

151. Measures of national income

152. Measuring well-being

153. Medium of exchange

154. Mental accounting

155. Menu costs

156. Mercantilism

157. Mergers and acquisitions

158. Minimum wage

159. Missing market

160. Model (economics)

161. Model (macroeconomics)

162. Modern portfolio theory

163. Monetarism

164. Monetary policy

165. Monetary reform

166. Money

167. Money supply

168. Monopoly profit

169. Moral hazard

170. Moral purchasing

171. Multiplier (economics)

172. National income

173. Natural Capitalism

174. Natural capital

175. Natural gross domestic product

176. Neo-Keynesian Economics

177. Neo-classical growth model

178. Neoclassical economics

179. Network effect

180. Network externality

181. *New Keynesian economics*
182. *New classical economics*
183. *Normal goods*
184. *Operations research*
185. *Opportunity cost*
186. *Output (economics)*
187. *Parable of the broken window*
188. *Pareto efficiency*
189. *Participatory economics*
190. *Petrocurrency*
191. *Poverty*
192. *Poverty level*
193. *Preference*
194. *Price*
195. *Price discrimination*
196. *Price elasticity of demand*
197. *Price points*
198. *Production, costs, and pricing*
199. *Production (economics)*
200. *Production function*
201. *Production theory basics*
202. *Productivism*
203. *Productivity (economics)*
204. *Profit (economics)*
205. *Profit maximization*
206. *Prospect theory*
207. *Public bad*
208. *Public choice theory*
209. *Public debt*
210. *Public good*

211. *Purchasing power parity*
212. *Rate of return pricing*
213. *Rational choice theory*
214. *Rational expectations*
215. *Rational pricing*
216. *Reaganomics*
217. *Real business cycle*
218. *Real versus nominal in economics*
219. *Recession*
220. *Regression analysis*
221. *Reserve currency*
222. *Returns to scale*
223. *Risk premium*
224. *Safe trade*
225. *Sales tax*
226. *Saving (economics)*
227. *Scarcity*
228. *Seven-generation sustainability*
229. *Slavery*
230. *Social capital*
231. *Social cost*
232. *Social credit*
233. *Social welfare*
234. *Socialism*
235. *Specialization (functional)*
236. *Stagflation*
237. *Standard of living*
238. *Stock exchange*
239. *Subsidy*
240. *Subsistence agriculture*

241. *Substitute good*

242. *Sunk cost*

243. *Supply-side economics*

244. *Supply and demand*

245. *Surplus value*

246. *Sustainable competitive advantage*

247. *Sustainable development*

248. *Sweatshop*

249. *Tariff*

250. *Tax*

251. *Tax, tariff and trade*

252. *Technostructure*

253. *Terms of trade*

254. *The Theory of Moral Sentiments*

255. *Time-based currency*

256. *Time preference theory of interest*

257. *Time value of money*

258. *Trade*

259. *Trade bloc*

260. *Trade pact*

261. *Trader Ethic*

262. *Transaction cost*

263. *Triple bottom line*

264. *Trust (sociology)*

265. *U.S. public debt*

266. *UN Human Development Index*

267. *Uneconomic growth*

268. *Unemployment*

269. *United States dollar*

270. *Utilitarianism*

271. *Utility*
272. *Utility Maximization Problem*
273. *Value-added tax*
274. *Value (economics)*
275. *Value added*
276. *Value of Earth*
277. *Value of life*
278. *Virtuous circle and vicious circle*
279. *Wage rate*
280. *Wealth*
281. *X-efficiency*
282. *Yen*
283. *Yield (economics)*
284. *Zero-sum*
285. *Zone pricing*

Actors and filmmakers

This is a list of 233 names of pages relating to actors and filmmakers, as listed in the two subsections "*Featured articles*" and "*Good articles*" on the English language *Wikipedia* page "*Wikipedia:WikiProject Actors and Filmmakers*". The list of terms was retrieved from the URL *http://en.wikipedia.org/wiki/Wikipedia:WikiProject_Actors_and_Filmmakers* on 14 October 2012.

View counts were downloaded from *http://stats.grok.se/* on 14 October 2012.

1. *Aaron Eckhart*
2. *Aaron Sorkin*

3. *Abbas Kiarostami*
4. *Al Pacino*
5. *Alan Dale*
6. *Ali Larter*
7. *Amanda Bynes*
8. *Amanda Seyfried*
9. *Amy Adams*
10. *André Morell*
11. *Andrea Corr*
12. *Andy Harries*
13. *Angelina Jolie*
14. *Anna May Wong*
15. *Anne Hathaway*
16. *Anthony Michael Hall*
17. *Ashley Tisdale*
18. *Austin Nichols*
19. *Barbara Hershey*
20. *Beatriz Michelena*
21. *Ben Affleck*
22. *Ben Daniels*
23. *Ben Stiller*
24. *Ben Thompson*
25. *Bernard Lee*
26. *Bette Davis*
27. *Brad Pitt*
28. *Bridget Moynahan*
29. *Britney Spears*
30. *Bruce Willis*
31. *Bryce Dallas Howard*
32. *Carey Mulligan*

33. *Casey Donovan*
34. *Chloë Sevigny*
35. *Christina Milian*
36. *Christine Hakim*
37. *Christine Langan*
38. *Cillian Murphy*
39. *Clint Eastwood*
40. *Dan Castellaneta*
41. *Dan Povenmire*
42. *Daniel Craig*
43. *Daniel Day-Lewis*
44. *Daniel Radcliffe*
45. *David Bowie*
46. *David Carradine*
47. *David Mitchell*
48. *David Morrissey*
49. *David Morse*
50. *David Schwimmer*
51. *Deborah Kerr*
52. *Dennis Rodman*
53. *Diane Keaton*
54. *Diane Lane*
55. *Dolph Lundgren*
56. *Dorothy Parker*
57. *Drew Barrymore*
58. *Drew Carey*
59. *Dwayne Johnson*
60. *Dylan and Cole Sprouse*
61. *Edward Norton*
62. *Emilie de Ravin*

63. *Eminem*

64. *Emma Watson*

65. *Eric Bana*

66. *Eric McCormack*

67. *Ethan Hawke*

68. *Eva Green*

69. *Evan Rachel Wood*

70. *Evanna Lynch*

71. *Ewan McGregor*

72. *Fann Wong*

73. *Faten Hamama*

74. *Fay Ripley*

75. *Forest Whitaker*

76. *Gene Wilder*

77. *Genelia D'Souza*

78. *George Bernard Shaw*

79. *Gethin Jones*

80. *Halle Berry*

81. *Hank Azaria*

82. *Harriet Frank, Jr.*

83. *Heather Chasen*

84. *Henri-Georges Clouzot*

85. *Hugh Grant*

86. *Ilaiyaraaja*

87. *Jack Warner*

88. *Jackie Chan*

89. *Jacob Pavlovitch Adler*

90. *Jada Pinkett Smith*

91. *Jaime King*

92. *Jake Gyllenhaal*

93. *James Cagney*
94. *James Franco*
95. *James L. Brooks*
96. *James McAvoy*
97. *James Nesbitt*
98. *James T. Aubrey, Jr.*
99. *James Whale*
100. *Jane Fonda*
101. *Janet Jackson*
102. *Jason Beghe*
103. *Jason Isaacs*
104. *Jay Chou*
105. *Jayne Mansfield*
106. *Jenna Haze*
107. *Jennifer Connelly*
108. *Jessica Alba*
109. *Jim Henson*
110. *Joan Crawford*
111. *John Barrowman*
112. *Jon Cryer*
113. *Jon Hamm*
114. *Jonathan Pryce*
115. *Joseph Barbera*
116. *Judy Garland*
117. *Julie Kavner*
118. *Junko Sakurada*
119. *KaDee Strickland*
120. *Kajol*
121. *Kamal Haasan*
122. *Kareena Kapoor*

123. *Karthi*
124. *Kate Beckinsale*
125. *Kate Bosworth*
126. *Kate Winslet*
127. *Katharine Hepburn*
128. *Keira Knightley*
129. *Kevin Spacey*
130. *Kim Ki-young*
131. *Kimberley Joseph*
132. *Kirsten Dunst*
133. *Kristen Bell*
134. *Kroger Babb*
135. *Kylie Minogue*
136. *Larry David*
137. *Laurence Fox*
138. *Leonardo DiCaprio*
139. *Leslie Nielsen*
140. *Lily Cole*
141. *Lina Leandersson*
142. *Lindsay Lohan*
143. *Liv Tyler*
144. *Lucille Ball*
145. *Maggie Grace*
146. *Maggie Gyllenhaal*
147. *Malin Åkerman*
148. *Mamoru Miyano*
149. *Mani Ratnam*
150. *Mariah Carey*
151. *Matt Damon*
152. *Matt Smith*

153. *Melissa Rosenberg*
154. *Michael J. Fox*
155. *Michael Jackson*
156. *Michael Sheen*
157. *Michelle Pfeiffer*
158. *Mike Bullen*
159. *Mila Kunis*
160. *Miles Fisher*
161. *Milla Jovovich*
162. *Miranda Otto*
163. *Nancy Reagan*
164. *Naomi Watts*
165. *Natalie Portman*
166. *Nate Parker*
167. *Ned Manning*
168. *Noël Coward*
169. *Paudge Behan*
170. *Paul Reubens*
171. *Penélope Cruz*
172. *Peter Sarsgaard*
173. *Phil Hartman*
174. *Pierce Brosnan*
175. *Preity Zinta*
176. *R. Madhavan*
177. *Rachel Bilson*
178. *Rachel McAdams*
179. *Rachel Nichols*
180. *Rachel Weisz*
181. *Rajinikanth*
182. *Ralph Bakshi*

183. *Rani Mukerji*
184. *Ranveer Singh*
185. *Reese Witherspoon*
186. *Reginald Tate*
187. *Rena Mero*
188. *Riya Sen*
189. *Robert Bathurst*
190. *Robert Benchley*
191. *Robert Downey, Jr.*
192. *Robert Rossen*
193. *Ronald Reagan*
194. *Ronnie Barker*
195. *Roseanne Barr*
196. *Rudolph Cartier*
197. *Rupert Grint*
198. *Ryan Gosling*
199. *Samuel L. Jackson*
200. *Satyajit Ray*
201. *Scarlett Johansson*
202. *Scout Taylor-Compton*
203. *Sean Bean*
204. *Sebastian Shaw*
205. *Seth MacFarlane*
206. *Seth Rogen*
207. *Shane Hurlbut*
208. *Shia LaBeouf*
209. *Shriya Saran*
210. *Sienna Guillory*
211. *Sjumandjaja*
212. *Spencer Tracy*

213. *Stacy Keibler*
214. *Stanley Holloway*
215. *Stephen Colbert*
216. *Steve Dodd*
217. *Sydney Newman*
218. *Taylor Lautner*
219. *Tessa Ludwick*
220. *Tetsuji Takechi*
221. *Tina Fey*
222. *Tina Turner*
223. *Tom Felton*
224. *Tom Hooper*
225. *Tom Welling*
226. *Ulrich Mühe*
227. *Verna Fields*
228. *Vidya Balan*
229. *Vikram*
230. *Vivien Leigh*
231. *William Hanna*
232. *Wim Umboh*
233. *Winona Ryder*

Data pre-processing

Data on views and edits of *Wikipedia* pages were pre-processed for analysis. Data on views of *Wikipedia* pages were retrieved from *http://stats.grok.se/*. Preliminary analysis of the data revealed some periods for which no page views were reported for any pages in the analysis and nearby periods where recorded page views were abnormally low across all pages, suggesting periods of system downtime. To exclude these days in a systematic

manner, we therefore removed days from our analysis where no page views were reported for any of the pages analysed, or where the total number of views across all pages in the data set had fallen to below 2 standard deviations of the mean daily total views. For each of the three data sets, 30 days were removed due to no page views being reported across all pages. For the company page data set, 12 days were removed due to the total number of page views falling below the 2 standard deviation threshold. For the financial topic data set, 20 days were removed for this reason, and for the actors and filmmakers data set, 5 days were removed. We then calculated the mean number of daily page views for each week, where excluded days did not contribute to this mean calculation. For all datasets, out of 229 weeks analyzed, the two weeks beginning on Monday 14th July 2008 and Monday 21st July 2008 did not have any data for page views. The mean for these weeks was recorded as a missing value.

Data on edits of *Wikipedia* pages were retrieved directly from the related *Wikipedia* revision history page, and aggregated to determine the mean number of daily edits for a week.

SUPPLEMENTARY FIGURE:

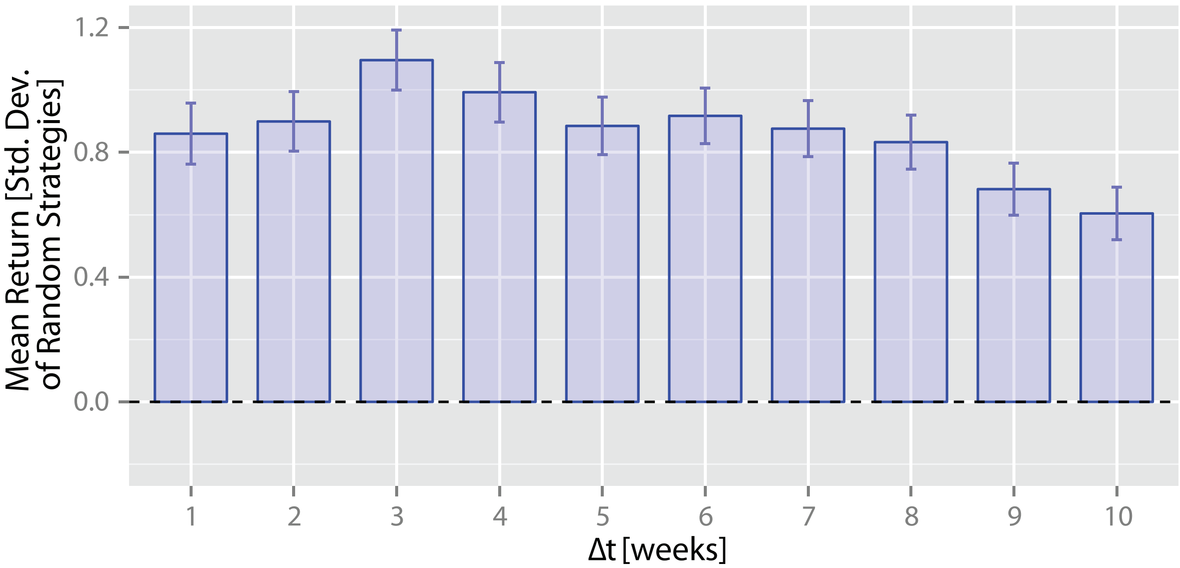

Figure S1. Effect of *Δt* on strategy returns. We investigate how the size of returns from the trading strategies based on changes in views of the 285 financially related *Wikipedia* pages is affected by *Δt*. The mean return from trading strategies in standard deviations of random strategies is shown for *Δt* = 1 to 10 weeks. Error bars show the standard error around the mean. Whilst the mean return of the strategies does differ significantly for different values of *Δt* ($\chi^2 = 93.26$, $df = 9$, $p < 0.001$; Kruskal-Wallis rank sum test), the mean return is greater than 0 at all values of *Δt* (all *W*s > 1950000, all *p*s < 0.001; all two-tailed two-sample Wilcoxon rank sum tests, using comparisons to the random strategy distribution for the whole period).
